# RETRACTED: Min et al. Malted Barley as a Potential Feed Supplementation for the Reduction of Enteric Methane Emissions, Rumen Digestibility, and Microbiome Community Changes in Laboratory Conditions. *Animals* 2025, *15*, 664

**DOI:** 10.3390/ani16050708

**Published:** 2026-02-25

**Authors:** Byeng Ryel Min, Uyeno Yutaka, Hossam Ismael, Heba Abdo, Santosh Chaudhary, Mariline Hilaire, Vivian Kanyi

**Affiliations:** 1 Department of Agricultural and Environmental Sciences, Tuskegee University, Tuskegee, AL 36088, USA; hismael@tuskegee.edu (H.I.); habdo@tuskegee.edu (H.A.); schaudhary8992@tuskegee.edu (S.C.); mhilaire6334@tuskegee.edu (M.H.); vkanyi1503@tuskegee.edu (V.K.); 2 Department of Agriculture, Shinshu University, Minamiminowa 8304, Nagano 3994511, Japan; ytkuyeno@shinshu-u.ac.jp

The journal retracts the article titled “Malted barley as a potential feed supplementation for the reduction of enteric methane emissions, rumen digestibility, and microbiome community changes in laboratory conditions” [1], cited above.

Following publication, concerns were brought to the attention of the Editorial Office regarding the unauthorized use of propriety data presented within this article [1].

Adhering to our standard procedure, the Editorial Office and Editorial Board conducted an investigation that confirmed that appropriate permission to publish the underlying data was not obtained as set out by USDA ARS. As retroactive permission could not be obtained, and at the request of the USDA ARS, the Editorial Board has decided to retract and remove this article as per MDPI’s retraction policy (https://www.mdpi.com/ethics#_bookmark30, accessed on 10 December 2025). As such, the title, author information and this retraction note will remain permanently available, and the article itself has been removed. This information has also been transmitted to relevant indexing organizations.

This retraction was approved by the Editor-in-Chief of the *Animals* journal.

The authors have agreed to this decision.
